# Effect of Educational Intervention on Adverse Drug Reporting by Physicians: A Cross-Sectional Study

**DOI:** 10.1155/2014/259476

**Published:** 2014-03-18

**Authors:** Manisha Bisht, Shruti Singh, D. C. Dhasmana

**Affiliations:** ^1^Department of Pharmacology, AIIMS Rishikesh, Virbhadra Road, Rishikesh, Uttarakhand 249 201, India; ^2^Himalayan Institute of Medical Sciences, Jolly Grant, Dehradun, Uttarakhand 248140, India

## Abstract

In India, the pharmacovigilance program is still in its infancy. National Pharmacovigilance Program of India was started for facilitating the pharmacovigilance activities. The ADR reporting rate is still below satisfactory in India. This cross-sectional questionnaire based study was carried out in a tertiary care teaching hospital in Uttarakhand, which is a peripheral ADR monitoring centre to assess the level of knowledge, attitude, and the practices of pharmacovigilance among the doctors and to compare it with the group of doctors attending educational CME for improving awareness of pharmacovigilance. The most important revelation of this study was that although adequate knowledge and the right attitude about adverse drug reaction reporting were instigated in the doctors after the educational intervention, the practice was still neglectful in both groups, emphasizing the need to design the strategies to develop adverse drug reaction reporting culture.

## 1. Introduction

Adverse drug reactions (ADRs) are among the significant cause of morbidity and mortality worldwide [1]. ADRs not only pose a risk to the patient's safety, but also adversely affect their quality of life and increase the healthcare cost considerably [2, 3]. ADR incidence has been reported to range from 5.9 to 22.3% of all emergency department admissions [4]. India has one of the largest drugs consuming population, with majority of people belonging to low socioeconomic group. Thus, it is the need of the hour to identify adverse drug reactions as early as possible and to prevent them if possible, for ensuring the well-being of the patient at reasonable cost. Pharmacovigilance, which relates with the detection, assessment, understanding, and prevention of ADR to medicines, is of utmost importance in this regard. In India, National Pharmacovigilance Program of India (PvPI) is responsible for conducting activities related to ADR monitoring. Spontaneous reporting of ADRs by health professionals is the corner stone of pharmacovigilance. The health professionals have major contribution in signal detection of unsuspected and unusual ADRs previously undetected during the initial evaluation of a drug [5]. The major limitation associated with spontaneous ADR reporting system is underreporting [6]. It is estimated that only 6–10% of all ADRs are reported [7]. India rates below 1% in terms of ADR reporting [8]. This clearly emphasises that the current status of pharmacovigilance in India is far from satisfactory. Pharmacovigilance has been included in the medical undergraduate and postgraduate curriculum in many medical colleges to instigate the pharmacovigilance program. Knowledge, attitude, and the practices (KAP) analysis may provide insight into the factors associated with underreporting of ADRs. Many studies have attributed inadequate knowledge about ADRs to that of underreporting of ADRs [9–11]. Therefore, increasing awareness about pharmacovigilance should be the first step to facilitate the reporting of ADRs. This study was a humble attempt taken in that direction and it was endeavoured to assess the level of knowledge, attitude, and the practices of pharmacovigilance among the doctors and to compare it with the group of doctors attending educational CME for improving awareness of pharmacovigilance.

## 2. Material and Methods

This cross-sectional questionnaire based study was conducted in a tertiary care teaching hospital in Uttarakhand, which is a peripheral ADR monitoring centre (AMC) since February 2011. In December 2011, a CME was conducted by the department of pharmacology for increasing the awareness of ongoing pharmacovigilance program in the institute, which were attended by some of the faculty members and residents. During this session, physicians and resident doctors were also encouraged to report all suspected ADRs, including those that were mild or anticipated. This KAP questionnaire survey was conducted during June 2011 to August 2011 and approval from institutional ethical committee was obtained prior to administering the questionnaire survey. The survey questionnaire was administered to 125 doctors who had attended the CME and 125 doctors who had not attended the CME.

The study tool was a predesigned questionnaire adapted from previous studies with some changes to adapt local conditions [9, 12]. The final KAP questionnaire consisted of 21 questions out of which question numbers 1 to 10, 11 to 16, and 17 to 21 were designed to specifically assess the knowledge, attitude, and practice, respectively, regarding adverse reaction reporting. In order to preclude any potential bias, the disclosure of name of the responder was made optional. For ensuring the response rate, all the doctors were provided the questionnaire personally and requested to fill it on the same day and the duly filled questionnaire was collected on the same day. Suggestions on the possible ways to improve the ADR reporting were welcome.

Chi-square test was used to compare the awareness, attitude, and practice of pharmacovigilance of the doctors who had attended the educational program with that of those who had not attended educational program to evaluate the impact of the effectiveness of educational intervention among healthcare professionals; the level of statistical significance was set at *P* < 0.05.

## 3. Results

Out of 250 faculty members and residents that approached to participate in the study, 200 completed and returned the questionnaire, giving the response rate of 80%. The survey questionnaire was analyzed question-wise and their percentage value was calculated.

### 3.1. Knowledge of Physicians

Table 1 compares the knowledge of the doctors who had attended the educational CME on pharmacovigilance with that of those who had not attended it. The results revealed that the doctors who attended the educational CME have significant increase in the knowledge regarding pharmacovigilance. More doctors in the CME group (68% versus 48%; *P* < 0.005) encountered ADRs during their clinical practice and were aware of drugs banned due to ADR. Almost all doctors in both groups revealed that they were qualified to report adverse reactions to drugs, while pharmacists and physiotherapists were the least considered to report an ADR. Only 43% of doctors in the non-CME group were aware of the existence of PvPI as compared to 93% of doctors in the CME group and this difference was highly significant statistically. Similarly, 81% of doctors in the CME group as compared to 36% in non-CME group were aware of the AMC in the institute. Most of the doctors in both groups were not aware that adverse effects due to herbal medications also have to be reported.

### 3.2. Attitude of Physicians

**Figure 1:**
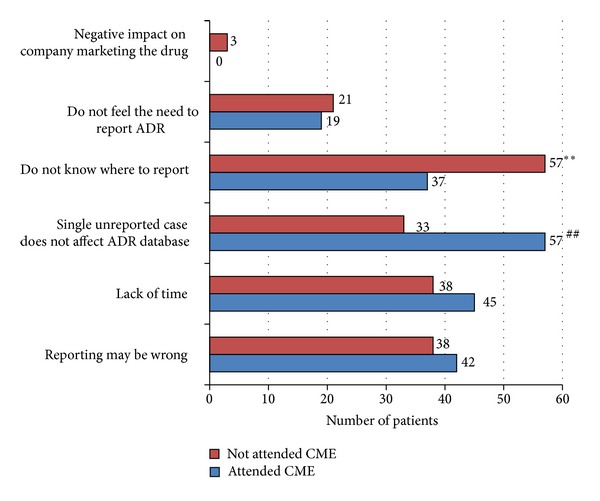
Graph showing the factors discouraging the ADR reporting among the physicians of both groups.

The attitude of the doctors who had attended the educational CME on pharmacovigilance with that of those who had not attended it is compared in Table 2. The main purpose of pharmacovigilance according to the doctors who had attended the CME was to identify predisposing factors to ADRs followed by the identification of safe drugs. The doctors in the other group (not attended the CME) recognised the identification of safe drugs and new ADRs as the main purpose of pharmacovigilance. In the CME group, most respondents were encouraged to report ADRs if the reaction was to a new product (65% versus 47%; *P* < 0.05), whereas in non-CME group the seriousness of the ADRs was the most important factor (94% versus 57%; *P* < 0.0001).  Figure 1 represents the major reasons for not reporting ADR by physicians. In the CME group, the notion that reporting of only one ADR makes no significant contribution to the ADR database (57% versus 33%; *P* < 0.0001) was the most important reason which discouraged the physician from ADR reporting. In contrast, unawareness of the reporting centre (57% versus 37%; *P* < 0.05) was the most important discouraging factor in ADR reporting among doctors in non-CME group. A good number of doctors in both groups (51%, 61%) were of the opinion that ADR reporting is a professional obligation for them while nearly one-third of patients in non-CME group did not consider ADR reporting as a professional obligation (28% versus 13%; *P* < 0.05). In CME group, majority of doctors opined that all serious ADRs should be reported (55% versus 39%; *P* < 0.05), whereas most doctors in non-CME group felt that all ADRs should be reported (56% versus 36%; *P* < 0.05). Majority of doctors in both groups (58%, 68%) opined that ADR reporting centre should be established in all the hospitals. Most of the doctors in both groups especially non-CME group felt that ADR reporting should be compulsory (48% versus 64%; *P* < 0.05).

### 3.3. Practice of Doctors

**Figure 2:**
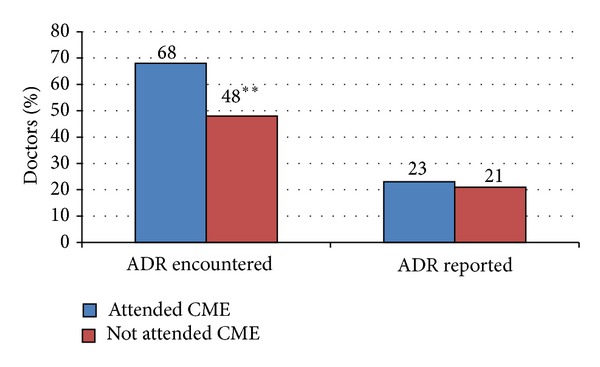
ADR observed and reported by physician of both groups.

The comparison of practice of the doctors who had attended the educational CME on pharmacovigilance with that of those who had not attended it is given in Table 3. Majority of doctors in the CME group conveyed that they updated their knowledge regarding ADRs of new drugs from scientific journals (69%), seminars (65%), and internet (63%), whereas doctors in non-CME group preferred sources like internet (49%), scientific journals (42%), and textbooks (40%) for updating their knowledge regarding ADRs of new drugs. Doctors in the CME group acknowledged more free access to ADR reporting form as compared to doctors in non-CME group (63% versus 19%; *P* < 0.0001). Interestingly, majority of doctors in both groups (77% versus 68%) did not report any ADR to date (Figure 2). Majority of doctors in the CME group admitted that the information on the ADR reporting form was clear as compared to the doctors in non-CME group (58% versus 14%; *P* < 0.0001). Most of the doctors in both groups felt the need for training on filling of ADR reporting form. Regarding the mode of reporting ADRs, the doctors in both groups preferred e-mail followed by direct contact. Both groups of doctors (CME group—56%; non-CME group—51%) mentioned that attending conferences and continuing medical education (CME) and other similar activities would facilitate the significance of PhV and majority of them emphasised the need to conduct such activities periodically (CME group—45%; non-CME group—43%). The doctors in the non-CME group emphasized more on making ADR reporting forms easily accessible and simplifying the process of reporting.

**Figure 3:**
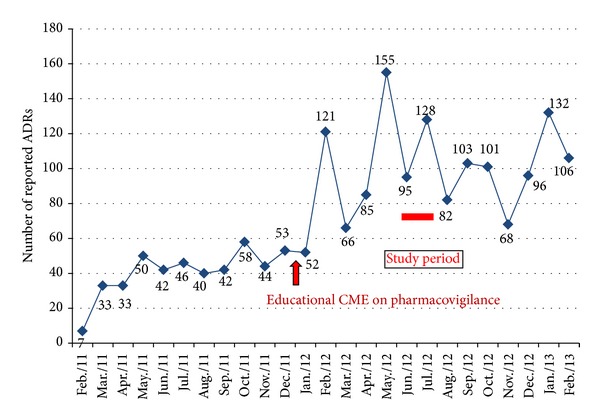
ADR reports in the AMC of the hospital between February 2011 and 2013.

Figure 3 depicts the total number of ADRs reported in the AMC of the tertiary care hospital between February 2011 and February 2013. Initially after the educational intervention, there was increase in number of ADR reports followed by decline in total number of reports.

## 4. Discussion

In India, the pharmacovigilance related activity was started initially in 1986 [13]. After initial futile attempts, Ministry of Health and Family Welfare, Government of India, relaunched this program as Pharmacovigilance Program of India (PvPI) in July 2010 [14]. Since then, continuous efforts are being made to strengthen the PvPI by including more and more MCI recognised medical colleges in this program. This study was conducted in a tertiary care teaching hospital after the pharmacovigilance program was started. It was a cross-sectional questionnaire based study, comparing the level of knowledge, attitude, and practice of pharmacovigilance between doctors who were educated on the subject of pharmacovigilance through a CME based educational program with that of other doctors who were not.

This study highlighted that significantly less number of doctors, who had not attended educational CME on pharmacovigilance, had the adequate knowledge of pharmacovigilance. Many studies conducted in India have also reported poor knowledge of doctors regarding pharmacovigilance [15, 16]. The doctors who had attended the CME on pharmacovigilance had increased awareness regarding the pharmacovigilance program and ADR monitoring. Similarly, previous studies have also confirmed that educational intervention leads to increased awareness regarding pharmacovigilance [12, 17]. Thus, continuous efforts are required for increasing the awareness of pharmacovigilance through the provision of appropriate education and training programmes at regular intervals for ADR reporting. Majority of doctors in both groups had encountered ADRs during their clinical practice. This was an encouraging finding as recognition of ADR is the first step of pharmacovigilance. However, other studies have reported higher number of doctors encountering ADRs during their clinical practice [16, 18]. This reflects that there is further need to enhance the knowledge of the doctors in the institute regarding ADR.

The most common discouraging factor for ADR reporting in doctors not educated on pharmacovigilance was the lack of knowledge on where to report indicating ignorance. This factor is adequately taken care of by increasing the awareness about existing pharmacovigilance centre, as reflected in our results where more than 60% of doctors in CME group were aware of as to where to report. On the other hand, most common notion of doctors in the CME group was that single unreported case does not affect ADR database reflecting indifferent attitude towards ADR reporting. Other studies have also reported similar attitudes regarding ADR monitoring [9]. The indifference in attitude regarding ADR reporting should be specifically addressed by emphasising the need to report all ADRs, as it significantly influences the ADR reporting among the doctors. The doctors in non-CME group were more willing to report to serious and unusual ADR but only one-third of doctors felt the need to report all ADRs. Whereas more than half of the doctors in CME group felt the need to report all ADR and preferred to report ADR with new drug or unusual side effect of old drugs. This reflects that educational intervention can emphasise the need to report all ADRs and doctors may be willing to report all ADRs if adequate knowledge is imparted to them. Moreover, doctors in both groups had a positive attitude towards ADR monitoring. Most of them opined that pharmacovigilance centre should be established in all the hospitals and ADR reporting should be compulsory. This indicates that doctors are keen to learn and practice pharmacovigilance if proper knowledge and training about ADRs reporting are imparted to them.

In this study, it was observed that more doctors in the CME group as compared to non-CME encountered ADRs during their clinical practice (68% versus 48%) but the number of doctors who had ever reported ADR was similarly inadequate in both groups (23% versus 21%). Similar trend in terms of ADR encountered and ADR reporting was observed in other studies also. Various studies have reported that though majority of doctors felt the need of ADR reporting and they frequently encounter ADRs during their clinical practice but most of them have never reported any ADR [9, 19, 20]. This reflects that even though awareness of ADR reporting can be increased by initial educational intervention, more effort is required to influence the practice of ADR reporting. In some countries like UK, France, The Netherlands, and Sweden, the ADR reporting rates are much higher ranging from 40 to 70% [9, 20–24]. The main reason for this may be that in these countries ADR monitoring system is well established and ADR reporting is mandatory.

The most important revelation of this study was that although adequate knowledge and the right attitude about ADR reporting were instigated in the doctors after the educational intervention, the practice was still neglectful in both groups. Though, initially, there was an increase in the number of ADR reports after the educational intervention, there was again a decline in the numbers after some time (Figure 3). Another study has also reported similar findings where an educational intervention improved physician awareness of ADRs and the same was incorporated into their everyday clinical practice [25]. However, similar to our study, the effects of the educational intervention were temporary.

Fostering a positive ADR reporting culture amongst the clinicians is indeed a difficult task. The awareness about pharmacovigilance should commence from the beginning and it should be incorporated in the medical teaching and training curriculum. Besides this, continuous education programmes should be carried out to emphasize the importance of ADR reporting and regular communication with clinician's should be carried out to explain the reporting procedures and thereby inculcate the habit of ADR reporting. As expected, lack of time was accounted as the second most discouraging factor for ADR reporting in both groups. Therefore, the most important factor to reinforce the pharmacovigilance is to provide an easy and quick method of reporting. Apart from this, the study participants also suggested that the acknowledgment of the receipt of the report and the outcome of the reporting helps in motivating them to continue the pharmacovigilance activities.

Previously, many studies have been carried out in India as well as abroad to assess the KAP of doctors regarding ADR monitoring [9, 11, 12, 17]. This study was a step further where we had tried to compare the impact of educational intervention on the KAP of pharmacovigilance in doctors. Most significant conclusion of our study was that even though educational intervention improves the knowledge regarding pharmacovigilance, its impact on practice is relatively transient. This study opens new avenues for further research for the assessment of the strategies that are helpful in improving the practice of pharmacovigilance.

## 5. Conclusion

Pharmacovigilance can survive only on the spontaneous reporting by the healthcare professionals, which in turn depends on their good knowledge about PhV as well as their willingness to report. Our study concludes that creating awareness among the healthcare professionals and promoting ADR reporting are the need of the hour. Organising regular workshops and continuous medical education will improve the awareness of healthcare professional regarding PV and other strategies have to be developed for facilitating the ADR reporting culture in our country.

## Figures and Tables

**Table 1:** Knowledge of the physicians of both groups regarding pharmacovigilance.

	Doctors attending CME	Doctors not attending CME
ADRs encountered per week		
None	32	52*
1–5/week	53	45
6–10/week	13	2*
>10/week	2	1
Correct definition of pharmacovigilance	58	24^#^
Health professional qualified to report ADR		
Medical doctors	99	96
Dental doctors	83	61^#^
Nurses	48	58
Pharmacists	47	44
Physiotherapists	32	15*
All healthcare professional	11	14
Doctors aware of pharmacovigilance programme of India	93	43^#^
Doctors aware of regional pharmacovigilance centre in HIHT	81	36^#^
Aware of any drug banned due to ADR	86	54^#^
Doctors not knowing the agents to be reported for the ADR		
Vaccine	20	21
Herbal medicines	79	70
Over-the-counter drugs	62	39*
Antibiotics	4	15
Topical agents	47	43

*P < 0.005; ^#^ *P* < 0.0001.

**Table 2:** Attitude of the physicians of both groups regarding pharmacovigilance.

	Doctors attending CME	Doctors not attending CME
Main purpose of ADR reporting system		
Identify safe drugs	25	42**
Measure the incidence of ADRs	33	17*
Identify predisposing factors to ADRs	26	8^##^
Identify new ADRs	17	22
Comparison of ADRs within the same class	8	11
Factors encouraging ADR reporting		
Seriousness of ADR	57	94^#^
Unusualness of ADR	59	53
New drug	65	47**
Correct diagnosis	12	21
Well-recognised ADR	24	36
Factors discouraging ADR reporting		
Reporting may be wrong	42	38
Lack of time	45	38
Single unreported case does not affect ADR database	57	33^#^
Do not know where to report	37	57**
Do not feel the need to report ADR	19	21
Negative impact on company marketing the drug	—	3
Is ADR reporting a professional obligation?		
Yes	51	61
No	13	28**
Do not know	29	8*
Perhaps	7	3
Which ADR should be reported?		
None	—	1
All	36	56**
All serious ADRs	55	39**
ADRs to new drugs	32	11^##^
Unknown ADRs to old drugs	7	7
Opinion regarding establishment of ADR reporting centre		
Should be in all hospitals	68	58
Not needed in all hospitals	11	8
One in a city	10	10
Depend on bed size	16	21
ADRs reporting should be		
Compulsory	48	64**
Voluntary	38	15^#^
Rewarded	2	9
Hide the identity of prescriber	6	6
Hide the identity of reporter	6	6

*P < 0.005; ^#^ *P* < 0.0001; **P < 0.05; ^##^ *P* < 0.001.

**Table 3:** Attitude of the physicians of both groups regarding pharmacovigilance.

	Doctors attending CME	Doctors not attending CME
Sources used to gather information about ADRs		
Textbooks	38	40
Journals	69	42*
Medical representatives	7	17**
Internet	63	49
Seminar/conferences	65	22^#^
Drug promotional literature	9	26*
ADRs reported till now		
None	77	68
1–5	15	25
6–10	1	7
15–20	4	—
	1	—
Free access to ADR reporting form	63	19^#^
Information was clear on the form	58	14^#^
Need for training on filling of ADR reporting form	90	82
Method preferred for reporting ADR information		
Direct contact	40	32
Telephone	26	25
Email	44	54
Self	15	7
Other	17	4*

*P < 0.005; ^#^ *P* < 0.0001; **P < 0.05; ^##^ *P* < 0.001.
